# Acral metastasis of colonic cancer: A case report

**DOI:** 10.5339/qmj.2026.15

**Published:** 2026-03-22

**Authors:** Haya Alkuwari, Alyaa AL-Wuhaili, Noora Al-Sulaiti, Hisham Osman, Rafif Mahmood Al Saady

**Affiliations:** 1 College of Medicine, QU Health, Qatar University, Doha, Qatar; 2 Department of Surgery, Al Ahli Hospital, Doha, Qatar *Email: rafif@qu.edu.qa

**Keywords:** Acrometastasis, adenocarcinoma, colorectal cancer, immunohistochemistry, metastasis

## Abstract

**Background:**

Colorectal cancer (CRC) is the third most common malignancy worldwide and accounts for 9.4% of all cancer-related deaths, making it the second leading cause of cancer mortality. Approximately 70% of patients develop metastatic disease, most commonly to the liver and lungs. Acral metastasis, particularly to the digits, is extremely rare and usually indicates advanced disease with a poor prognosis.

**Case presentation:**

We report a rare case of digital acrometastasis from colorectal adenocarcinoma in a 55-year-old male. The patient presented with a painful lesion on his right index finger, characterized by discoloration, a wound cavity, and granulation tissue at the fingertip. The patient had a prior history of treated colon cancer and was receiving immunotherapy. He underwent wedge excision and nail avulsion of the affected finger. Histopathological examination confirmed metastatic adenocarcinoma with an immunohistochemical profile consistent with colorectal origin. The patient was referred to the National Center for Cancer Care and Research (NCCCR); however, no follow-up information was available, as he did not return for further evaluation.

**Discussion:**

Bone metastasis from CRC is uncommon, and acrometastasis to the digits occurs in only 0.007–0.2% of cases. The differential diagnosis of digital lesions can be challenging and should include both benign and malignant conditions. Immunohistochemical staining is crucial for diagnosis, and with metastatic CRC typically expressing cytokeratin 20 (CK20) and caudal type homeobox (CDX2), it helps distinguish it from primary digital adenocarcinomas. Clinicians should consider acrometastasis in patients with a history of CRC who present with unexplained digital lesions.

**Conclusion:**

This case underscores the rare potential of CRC to metastasize to acral regions, including the distal phalanges. Clinicians should maintain a high index of suspicion for metastatic disease when evaluating digital lesions in patients with a history of malignancy, as timely diagnosis and management are essential for optimizing patient outcomes.

## 1. INTRODUCTION

According to the GLOBOCAN database, colorectal cancer (CRC) is the third most commonly diagnosed cancer in men and the second most diagnosed cancer in women.^1^ In 2020, CRC accounted for 10% of all cancer cases and 9.4% of all cancer-related deaths worldwide. with a projected 3.2 million new cases globally by 2040.^2^ Significant regional differences in CRC incidence have been noted, with higher rates in Australia, Europe, and North America, compared to lower rates in South–Central Asia and Africa. In countries such as Japan, South Korea, and several Gulf Cooperation Council (GCC) nations—including Saudi Arabia, Oman, Yemen, the United Arab Emirates, Bahrain, Qatar, and Kuwait—as well as Slovakia, CRC represents the most frequently diagnosed malignancy among men.^3^ The majority of CRC-related mortality is attributable to metastatic disease, with approximately 22% of patients exhibiting metastases at the time of initial diagnosis.^4^ A previous study indicated that as many as 70% of patients with CRC may ultimately experience metastatic disease or relapse, with liver metastases occurring synchronously or metachronously in up to half of these cases.^1^ Studying the epidemiology of metastatic CRC is difficult, as most cancer registries do not systematically record data on the specific sites of metastasis.^5^

Colon cancer has been extensively studied due to its increasing incidence and well-defined risk factors, such as age, lifestyle, and genetic predisposition.^6^ Both hereditary factors and medical comorbidities, such as inflammatory bowel disease, also contribute to CRC risk.^7,8^ CRC arises through well-described pathways, including the adenoma–carcinoma pathway and microsatellite instability.^9^ Colon carcinoma can disseminate through both hematogenous and lymphatic pathways.^10^ CRC spreads hematogenously through the invasion–metastasis cascade and via lymphatics to regional lymph nodes, a process linked to E-cadherin downregulation—a cell adhesion molecule that normally maintains intercellular junctions.^10,11^ Approximately 10–15% of patients with CRC develop bone metastases, which are linked to processes such as epithelial–mesenchymal transition (EMT) and mesenchymal–epithelial transition (MET). These mechanisms enable tumor cells to detach, intravasate, and spread via the bloodstream to skeletal tissue.^12^ Metastasis to the limbs is rarer compared to the axial skeleton, with distal limbs less frequently involved than proximal limbs. Acral metastasis, defined as spread distal to the elbow or knee, usually reflects advanced systemic disease and may occur either after cancer diagnosis or, in up to 10% of cases, as the initial manifestation of malignancy.^13^

In this study, we report a case of colon cancer with acrometastasis to a digit. The patient was managed at Al Ahli Hospital, one of the largest private hospitals in Qatar, offering high-quality healthcare services with advanced medical technology.^14^ Qatar’s healthcare system is highly developed, with both public and private sectors.^15^

## 2. CASE PRESENTATION

**Figure 1.**
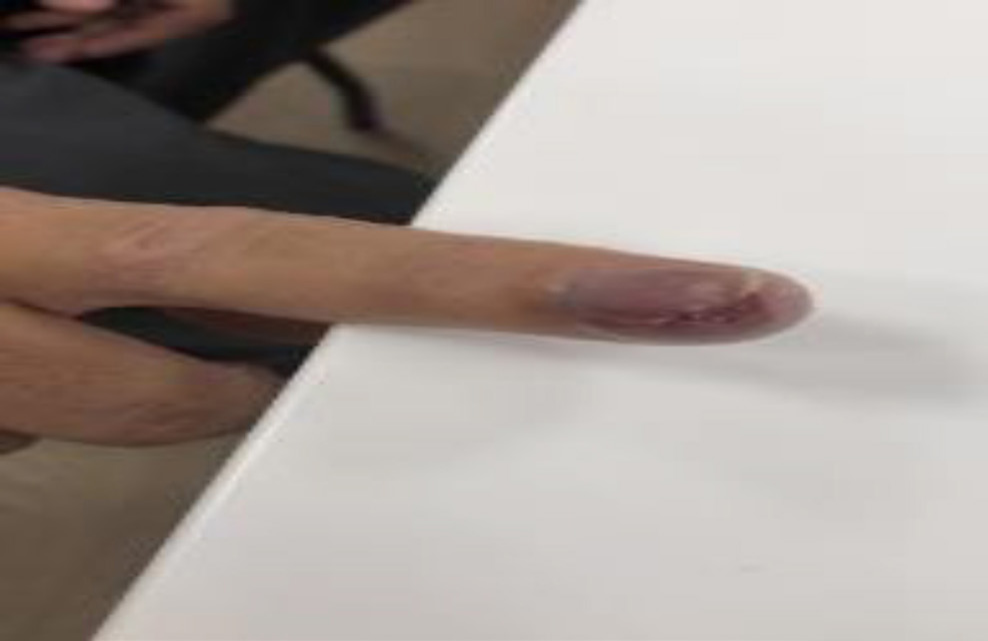
Right index finger with a wound and subungual purple pigmentation.

**Figure 2.**
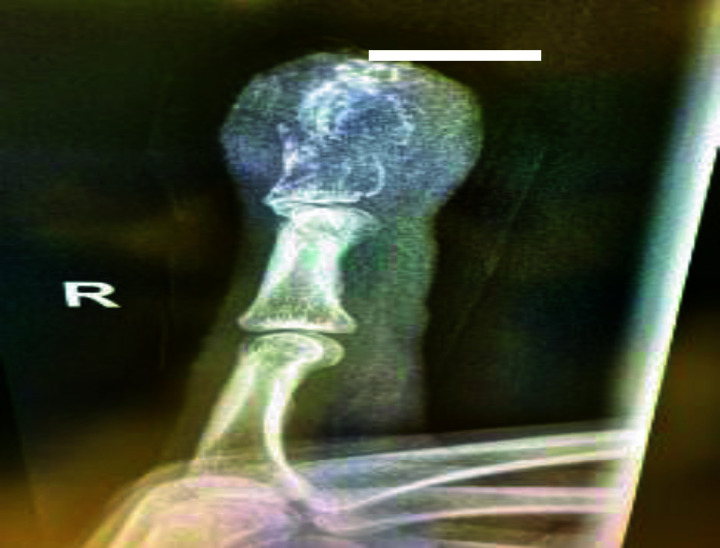
Plain X-ray of right index finger showing an ill-defined lytic lesion.

A 55-year-old male patient presented to the surgery clinic at Al Ahli Hospital with a painful lesion on his right index finger of a few weeks’ duration. He had no history of trauma or accident. The patient is married, a non-smoker, and a non-alcoholic. He had a history of treated colon cancer and was on immunotherapy, although details regarding the specific regimen and duration were not available. He had no known drug allergy. His family history was unremarkable, with no relevant genetic or psychosocial information. Physical examination showed a swollen right index finger with a wound cavity and subungual purple pigmentation, accompanied by granulation tissue at the fingertip (Figure 1). No other abnormalities were noted. A bacterial culture swab taken from the wound showed no growth, and an X-ray of the right index finger revealed an ill-defined lytic lesion involving the tuft of the distal phalanx with overlying cortical destruction, raising the suspicion for a focal bony lesion (Figure 2).

**Figure 3.**
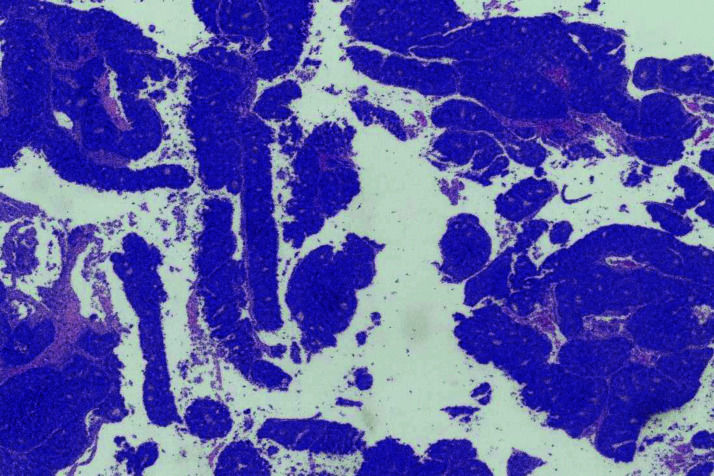
Microscopic appearance of the nailbed tissue (Hematoxylin and Eosin 400X).

**Figure 4.**
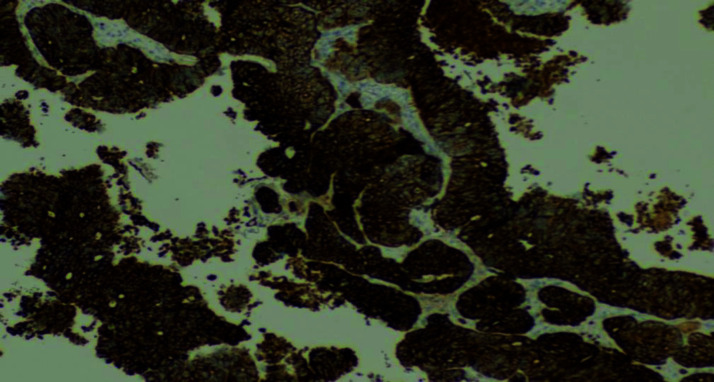
Immunohistochemical staining of the patient’s nailbed lesion showing CK20 positivity (400X).

**Figure 5.**
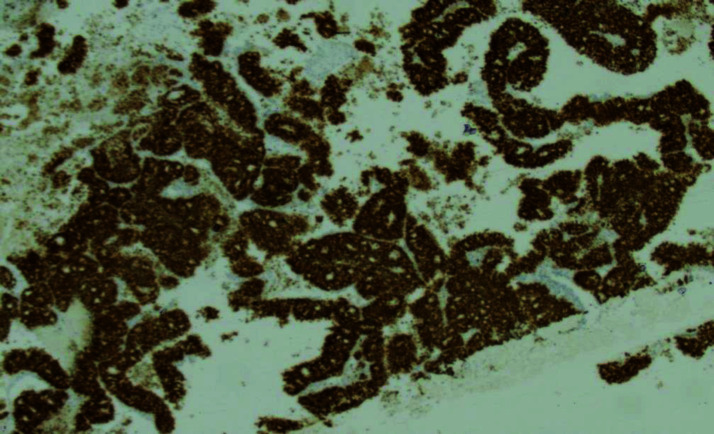
Immunohistochemical staining of the patient’s nailbed lesion showing CDX2 positivity (400X).

Wedge excision and avulsion of the right index nail were performed, and the nailbed tissue was sent for pathological examination. Histopathological analysis revealed malignant tumor fragments with glandular differentiation (Figure 3). The tumor cells were positive for cytokeratin 20 (CK20) and caudal type homeobox 2 (CDX2) immunostaining (Figures 4 and 5), and negative for cytokeratin 7 (CK7) immunostaining. Given the patient’s previous history of colon cancer, a diagnosis of metastatic colon cancer to the digit was made. The patient was referred to the National Center for Cancer Care and Research (NCCCR), the public cancer hospital in Qatar, which is part of Hamad Medical Corporation (HMC). Unfortunately, no follow-up information was available at Al Ahli Hospital, as the patient was an expatriate and did not return for further evaluation.

## 3. DISCUSSION

Approximately 25–30% of patients present with synchronous metastases of CRC at diagnosis, while up to 50% develop metastases during the course of their disease.^16^ CRC commonly metastasizes to several sites, with the liver and lung being the most frequent.^17^ Liver metastases are the most prevalent, affecting at least 25–50% of patients during disease progression,^18^ followed by lung metastases in 10–15% of cases.^19^ Other less common sites of metastasis include the peritoneum, found in approximately 8–15% of patients with CRC, and associated with a worse prognosis than metastases at other sites.^20^ Patients with CRC have a 3–7% risk of bone metastases.^21^ Para-aortic lymph node (PALN) metastases occur in 1–2% of CRC patients.^22^ Metastasis to the bone is considered rare, with most cases occurring in the vertebral column. Only 0.007–0.2% of the CRC metastasis are reported in the literature to involve the hand and wrist. In general, bone acrometastasis is associated with a poor prognosis.^23^ Other rare sites of metastasis from colorectal adenocarcinoma include the oral cavity.^24^ One reported case involved an 81-year-old man who presented with right wrist pain after complete remission of colon adenocarcinoma; a biopsy revealed glandular tissue consistent with metastatic colon adenocarcinoma.^23^ Another case described a 59-year-old female with a history of rectal adenocarcinoma who presented with a skin lesion—a ball of erythematous, crusted tissue on her left index finger—which was confirmed as metastatic disease.^25^ A 54-year-old African American male initially presented with swelling of his right middle finger; biopsy revealed mucinous adenocarcinoma, and subsequent colonoscopy confirmed CRC.^26^ On the other hand, digital soft tissue masses may also raise suspicion for benign lesions such as ganglia, giant cell tumors of the tendon sheath, and epidermal inclusion cysts.^27^ Felon is another common differential diagnosis for digital masses.^28^ Other malignancies that can present as digital masses include esophageal squamous cell carcinoma and bronchogenic carcinoma.^29^

Histopathologically, the differential diagnosis also included the possibility of a primary adenocarcinoma of the digit, such as aggressive digital papillary adenocarcinoma (ADPAC).^28^ Immunohistochemical staining plays a crucial role in distinguishing primary digital tumors from metastatic lesions. Metastatic colorectal adenocarcinomas commonly express CK20 and CDX2, whereas ADPAC may exhibit different staining patterns.^30^ A recent case report and literature review highlight the immunohistochemical profile of ADPAC, including vimentin, high molecular weight cytokeratin (HMWCK), and D2-40 for basaloid myoepithelial cells, and CK7 and epithelial membrane antigen (EMA) for the luminal/columnar cells.

The tumor cells also showed positivity for cytokeratin AE1/AE3, a combination of monoclonal antibodies including MOC31 and the antihuman epithelial antigen BEREP4, but were negative for both S100 protein and carcinoembryonic antigen (CEA).^31^

## 4. CONCLUSION

In conclusion, this case describes acral metastasis of colon adenocarcinoma to the distal phalanx of the right index finger, which has not been previously reported in the literature. Despite its rarity and infrequent presentation, acral metastasis should be considered in patients with a history of CRC who present with a suspicious digital lesion. The diagnosis was confirmed through histopathological examination, demonstrating glandular differentiation and specific immunohistochemical staining patterns consistent with a colorectal adenocarcinoma primary. The successful identification and management of this rare form of metastasis emphasize the need for vigilance and comprehensive evaluation in patients with a known history of CRC.

## COMPETING INTERESTS

The authors have no conflicts of interest to declare.

## AUTHORS’ CONTRIBUTION

HA, AA-W, NA-S: Analysis and writing. HO: Data acquisition and revision. RA: Study concept and design, data acquisition, editing, and revision.

## CONSENT

The patient provided verbal and written informed consent for publication of this case report.
